# *In Vivo* and *In Silico* Analysis of Quercetin’s Effects on Glycemic Regulation

**DOI:** 10.1155/sci5/5159975

**Published:** 2026-03-01

**Authors:** Jumriani Jumriani, Muhammad Aswad, Ratnawati Ratnawati, Filmaharani Filmaharani, Anggun Nurhidayah, Muhammad Rayza Azmin, Alfreds Roosevelt, Rizky Alfiana, Widya Hardiyanti, Nadila Pratiwi Latada, Mukarram Mudjahid, Firzan Nainu

**Affiliations:** ^1^ Postgraduate Program in Pharmacy, Faculty of Pharmacy, Hasanuddin University, Tamalanrea, Makassar, 90245, Indonesia, unhas.ac.id; ^2^ Department of Pharmaceutical Science and Technology, Faculty of Pharmacy, Hasanuddin University, Tamalanrea, Makassar, 90245, Indonesia, unhas.ac.id; ^3^ Unhas Fly Research Group, Faculty of Pharmacy, Hasanuddin University, Tamalanrea, Makassar, 90245, Indonesia, unhas.ac.id; ^4^ Department of Pharmacy, Sandi Karsa Polytechnic, Makassar, 90245, Indonesia; ^5^ Study Program of Pharmacy, Faculty of Medicine and Health Sciences, Universitas Muhammadiyah Makassar, Makassar, 90221, Indonesia, upm.edu.my; ^6^ Department of Pharmacy, Faculty of Pharmacy, Hasanuddin University, Tamalanrea, Makassar, 90245, Indonesia, unhas.ac.id

**Keywords:** diabetes mellitus, DPP4 inhibition, *Drosophila melanogaster*, molecular docking, quercetin

## Abstract

**Background & Objective:**

Diabetes mellitus is a global health issue caused by chronic hyperglycemia. Although various therapeutic options are available, each carries potential side effects, prompting growing interest in exploring natural compounds as alternative treatments. Quercetin, a flavonoid known for its antioxidant and anti‐inflammatory properties, is suspected to play a role in glucose regulation, although its molecular mechanisms remain incompletely understood. This study aimed to analyze the *in vivo* effects of quercetin on the phenotype of *Drosophila melanogaster* and to validate its potential mechanism through an *in silico* molecular docking approach, focusing on its interaction with diabetes‐related enzyme targets.

**Methods:**

Phenotypic evaluation included measurements of body morphology, locomotor activity, survival rate, and hemolymph glucose levels. Molecular analyses were conducted using reverse transcription quantitative PCR (RT‐qPCR), while molecular docking studies were performed to assess quercetin’s interaction with the enzyme dipeptidyl peptidase‐4 (DPP4).

**Results:**

Quercetin significantly reduced hemolymph glucose levels in both larvae (*p* < 0.0001) and adult flies (*p* < 0.001) within the concentration range of 1–10 μm without affecting adult flies’ locomotor activity or survival. Additionally, quercetin enhanced the expression of genes involved in metabolic and stress response and improved growth parameters and motor activity in larvae subjected to a high‐sugar diet. Molecular docking studies revealed that quercetin has a high affinity for DPP4, supporting its proposed hypoglycemic mechanism.

**Conclusion:**

This study provides both phenotypic and molecular evidence that quercetin exerts hypoglycemic effects in *D. melanogaster*, potentially mediated through DPP4 inhibition and modulation of metabolic and stress‐response pathways. These findings offer new insight into the mechanisms of quercetin in glucose regulation.

## 1. Introduction

Over the past two decades, the global incidence of hyperglycemia has increased significantly and is projected to rise further by 2050 [1, 2]. Persistent hyperglycemia is a hallmark of diabetes mellitus (DM), a chronic metabolic disorder caused by impaired insulin production, reduced cellular sensitivity to insulin, or a combination of both factors [3]. In 2021, the global prevalence of diabetes among individuals aged 20–79 years was estimated at 10.5% (536.6 million people) and is expected to reach 12.2% (783.2 million people) by 2045 [4].

Chronic hyperglycemia in DM is often accompanied by other metabolic disturbances that can lead to organ damage, severe complications, and life‐threatening conditions [5]. While diabetes management strategies include lifestyle modifications, dietary adjustments, oral antidiabetic medications, and insulin therapy in severe cases, long‐term pharmacological intervention is often necessary [5]. Although lifestyle changes can improve glycemic control, they are frequently insufficient as standalone measures. Additionally, barriers such as drug resistance, side effects, and high medication costs hinder sustainable diabetes management [6]. In response to these challenges, plant‐derived medicines have garnered increasing interest due to their affordability and accessibility. Although phytochemicals are often regarded as safer alternatives, emerging evidence suggests that some natural compounds can also pose toxicity risks, underscoring the importance of rigorous safety assessment. One such phytochemical with antidiabetic potential is quercetin [6].

Quercetin (3,5,7‐trihydroxy‐2‐(3,4‐dihydroxyphenyl)‐4H‐chromen‐4‐one) is a natural flavonoid found abundantly in a variety of plants, fruits, and vegetables, including onions, apples, berries, nuts, seeds, bark, flowers, and tea [7]. Pharmacological studies have demonstrated that quercetin provides numerous health benefits, including cardioprotective, anti‐inflammatory, anticancer, and antidiabetic properties [7–9]. Quercetin’s hypoglycemic activity is attributed to mechanisms such as enhancing insulin sensitivity, stimulating glycogen synthesis, and inhibiting α‐glucosidase activity [7]. Additionally, quercetin inhibits the dipeptidyl peptidase‐4 (DPP4) enzyme, thereby extending the activity of glucagon‐like peptide‐1 (GLP‐1) and gastric inhibitory polypeptide (GIP) hormones [6]. These multifaceted properties highlight quercetin’s potential as a candidate for antidiabetic drug development. However, to ensure safety and efficacy, pharmacokinetic and pharmacodynamic studies are required, typically using animal models [10]. In line with ethical considerations and the principles of reducing mammalian use as disease models, alternative animal models are increasingly being explored [11].

*Drosophila melanogaster* has emerged as an alternative model for drug screening and research due to its small size, ability to breed in large numbers under controlled conditions, and short life cycle, which enables rapid data collection and cost‐effective experimentation [12]. In recent decades, diabetes models in this species have been developed to study the molecular mechanisms of diabetes and evaluate potential therapeutic agents [12]. For example, a hyperglycemic model can be induced in larvae by feeding a high‐sugar diet (HSD), recapitulating key aspects of diabetes, including elevated blood glucose levels and metabolic dysfunction [13]. In *Drosophila*, insulin‐producing cells (IPCs) share functional similarities with pancreatic β‐cells in humans [13], making the fly a relevant model for studying glucose regulation and therapeutic development. Recent studies also highlight the utility of *D. melanogaster* in phytochemical and developmental research, including investigations on curcumin’s pharmacological effects and the role of phagocytic receptors in larval growth [14, 15]. Collectively, these strengths establish *D. melanogaster* as a powerful platform for investigating metabolic dysregulation and evaluating bioactive compounds such as quercetin.

Although quercetin has been evaluated in mammalian models and *D. melanogaster* for its antidiabetic potential, studies on the mechanistic basis of its antihyperglycemic effects in *in vivo* hyperglycemic models remain limited. Previous research primarily used noninduced flies, overlooking critical molecular and developmental differences. Furthermore, key genes involved in metabolic and stress responses, such as *dilp2*, *dilp3*, and *dilp5* (insulin‐like peptides regulating glucose homeostasis) [16], *Thor* (homolog of 4E‐BP, a downstream effector of FOXO involved in insulin and nutrient signaling) [17–19], *srl* (*spargel*, the PGC‐1*α* homolog regulating mitochondrial biogenesis and energy metabolism) [20], *totA* (*Turandot A*, a stress‐response marker) [21, 22], and *hsp70* (a heat‐shock protein associated with proteotoxic and cellular stress) [23], have not been comprehensively analyzed in this context. To address these gaps, the present study investigates the hypoglycemic effects of quercetin in *D. melanogaster* using both hyperglycemic larvae induced with an HSD and untreated adult flies. The study examines glucose levels, developmental metrics, and the expression of genes critical to metabolic and stress response. This research aims to provide a mechanistic understanding of quercetin’s effects while establishing a foundation for high‐throughput screening (HTS) of hypoglycemic compounds using *D. melanogaster* as a model organism.

## 2. Materials and Methods

### 2.1. Materials

Quercetin (CAS RN: 117‐39‐5, Product No. Q0112) was procured from Tokyo Chemical Industry (TCI), Tokyo, Japan.

### 2.2. Drosophila Stocks

The experimental organism utilized in this study was *Drosophila melanogaster* (*w* ^1118^), maintained and reared in vials containing solid media (food) at a controlled temperature of 25°C and 60% relative humidity with alternating light–dark cycles, each lasting 12 h (Figure 1(a)). Adult flies used for glucose assays were also randomly chosen from treatment cohorts before homogenization. For each replicate, 30 flies (18 females and 12 males) were used for mating to produce larvae for subsequent experiments. For all larval experiments (morphometrics, body weight, crawling, and hemolymph measurements), third‐instar larvae were randomly selected from treatment vials to minimize sampling bias (Figure 1(b)).

Figure 1Schematic overview of the experimental design employed in this study, illustrating experiments conducted in (a) adult flies and (b) third‐instar larvae. Que, Quercetin.

**Figure:**
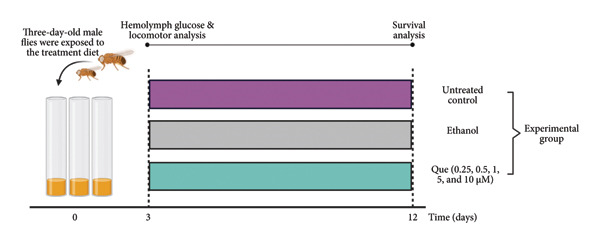
(a)

**Figure:**
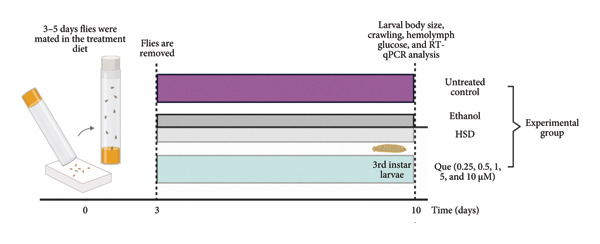
(b)

### 2.3. Preparation of Quercetin Solutions

Quercetin solutions were prepared using 70% ethanol as the solvent. The solutions were diluted to final concentrations of 0.25 μM, 0.5 μM, 1 μM, 5 μM, and 10 μM.

### 2.4. Preparation of HSD Food

HSD food for *D. melanogaster* was formulated by mixing cornmeal, yeast, agar, and 30% sucrose [24]. Subsequently, quercetin solutions at concentrations of 0.25 μM, 0.5 μM, 1 μM, 5 μM, and 10 μM were incorporated into the mixture.

### 2.5. Measurement of Larval Body Length, Width, and Weight

Third‐instar larvae were collected, washed with 0.9% NaCl to remove residual food, and dried with tissue paper. Body length and width were measured using a caliper. For body weight, larvae were transferred individually into pre‐weighed microcentrifuge tubes, and weights were determined to calculate the average body weight. Each assay was performed using five larvae per replicate, with five biological replicates per group, to ensure accuracy and reproducibility [25].

### 2.6. Crawling Assay

Crawling behavior was assessed in third‐instar larvae. Each larva (five per replicate) was placed on a Petri dish containing agar positioned on graph paper and allowed to crawl for 1 min. The crawling data were analyzed and visualized using GraphPad Prism software, facilitating muscle function and locomotor activity comparisons across experimental groups [26, 27].

### 2.7. Measurement of Larval Hemolymph Glucose

Third‐instar larvae were collected, washed with 0.9% NaCl, and dried with tissue paper. Hemolymph was extracted from approximately 70 larvae per sample by transferring them into Microcentrifuge tubes. The extracted hemolymph was centrifuged for 2 min, and 10 μL of the supernatant was mixed with 1000 μL of glucose reagent (GOD‐PAP) (Glory Diagnostics, Barcelona, Spain). After a 10‐min incubation, glucose levels were quantified via a UV–vis spectrophotometer (Shimadzu, Shimadzu Corp., Kyoto, Japan) at a wavelength of 500 nm [28].

### 2.8. Measurement of Hemolymph Glucose in Adult Flies

Hemolymph glucose levels were measured in 3‐day‐old adult flies maintained on either standard or treatment food. Three biological replicates were prepared for each control and experimental group, with each replicate comprising 10 mg of male flies (15–16 individuals). The flies were homogenized in 100 μL of PBS buffer, and the homogenate was centrifuged at 4°C for 2 min. Subsequently, 10 μL of the supernatant was mixed with 1000 μL of glucose reagent (GOD‐PAP) in a microcentrifuge tube. Absorbance was then measured at 500 nm using a UV–vis spectrophotometer [29].

### 2.9. Survival Assay

Survival rates of *w* ^1118^ flies were monitored from the initial day of quercetin treatment. Food was replaced every 3 days, and mortality rates within each vial were recorded daily throughout the experimental period [24].

### 2.10. Negative Geotaxis Analysis

The locomotor ability of each treatment group was evaluated using negative geotaxis analysis. Flies were transferred into sterile vials (Biologix, China) and allowed to acclimate for 1 min before testing. The vials were gently tapped on a table to ensure all flies started at the bottom, and they were then given 15 s to climb. The number of flies exceeding an 8 cm mark was recorded for each trial [30, 31].

### 2.11. Gene Expression Analysis

A total of 10 *w* ^1118^ larvae from each treatment group were collected for RNA isolation using the PureLink RNA Mini Kit (Invitrogen, Thermo Fisher Scientific Inc., Carlsbad, USA), following the manufacturer’s protocol. Gene expression levels were analyzed via real‐time reverse transcription PCR (RT‐qPCR) using the Universal One‐Step RT‐qPCR Kit (Luna, New England Biolabs, Inc., U.S.). RT‐qPCR reactions were performed with gene‐specific primers (Table 1) in a 10‐μL reaction volume. The thermal cycling conditions were as follows: an initial step at 50°C for 10 min, followed by 95°C for 2 min, and 40 cycles of 95°C for 10 s, 60°C for 30 s, and 72°C for 30 s. A standard melt curve analysis was conducted to confirm the specific amplification of the expected product. Expression levels of target genes were normalized to the ribosomal gene *rp49*, which served as an internal control.

**Table 1:** Primers used in RT‐qPCR analysis.

Genes	Forward (5′‐3′)	Reverse (5′‐3′)
*dilp2*	TCT​GCA​GTG​AAA​AGC​TCA​ACG​A	CAA​CTG​CAG​GGG​ATT​GAG​G
*dilp3*	ATC​CTT​ATG​ATC​GGC​GGT​GT	GTT​CAC​GGG​GTC​CAA​AGT​TC
*dilp5*	CCC​CGC​CTT​GAT​GGA​CAT​G	CAT​GTG​GTG​AGA​TTC​GGA​GCT​A
*srl*	CTCTTGGAGTCCGAGATCCGCAA	GGGACCGCGAGCTGATGGTT
*totA*	CCCTGAGGAACGGGAGAGTA	CTTTCCAACGATCCTCGCCT
*Thor*	AAGCAGACCAAGTCGCTGAA	ATTTGGCAGTTGCTGCATGG
*hsp70*	AGC​CGT​GCC​AGG​TTT​G	CGT​TCG​CCC​TCA​TAC​A
*rp49*	GAC​GCT​TCA​AGG​GAC​AGT​ATC​TG	AAA​CGC​GGT​TCT​GCA​TGA​G

### 2.12. Molecular Docking

Ligand structures were retrieved from the PubChem database (National Center for Biotechnology Information, NCBI), specifically quercetin (CID: 5280343) and vildagliptin (CID: 6918537). The crystal structure of DPP4 (PDB ID: 2P8S) was retrieved from the Protein Data Bank. Molecular docking was performed using UCSF‐Chimera integrated with AutoDock Vina 1.16. Ligand and protein structures were prepared with the Dock Prep tool in UCSF‐Chimera. The docking procedure involved defining a grid box covering the active site of DPP4 (center: *x* = 40.7113, *y* = 51.4588, *z* = 36.0376; dimensions: *x* = 15.4174, *y* = 9.31098, *z* = 6.35071). The exhaustiveness parameter was set to 8. Validation was carried out by redocking the cocrystallized ligand, which yielded an RMSD < 2 Å, and is considered to be an excellent re‐docking [32]. The docking results were visualized and analyzed with Discovery Studio 19.1 [33, 34].

### 2.13. Data Analysis

Data were analyzed using GraphPad Prism 9. One‐way ANOVA with Dunnett’s post hoc test was applied, and significance was set at *p* < 0.05. Locomotor data were analyzed using two‐way ANOVA. Results are presented as mean ± SD.

## 3. Result

### 3.1. Quercetin Improves Body Length, Width, and Weight and Enhances Crawling Activity in Hyperglycemic Larvae

This experiment aimed to assess the effects of an HSD on growth parameters, including body size (length and width), body weight, and larval motor activity. Additionally, the study explored the potential protective role of quercetin in mitigating the adverse effects associated with hyperglycemic conditions in *D. melanogaster* larvae. HSD caused significant growth retardation and reduced locomotor performance in *D. melanogaster* larvae. Compared with the control group, HSD‐fed larvae showed markedly reduced body length (Figure 2(a)), body width (Figure 2(c)), body weight (Figure 2(e)), and crawling speed (Figure 2(g)).

Quercetin supplementation attenuated these detrimental effects in a dose‐dependent manner. Larvae treated with quercetin exhibited significantly increased body length (Figure 2(b)), body width (Figure 2(d)), and body weight (Figure 2(f)) compared with HSD‐fed larvae. Locomotor activity also improved following quercetin exposure, with the most pronounced recovery observed at 10 μM (Figure 2(h)).

Figure 2Improvement in body length, width, weight, and crawling analysis following quercetin administration in *D. melanogaster* larvae. (a, b) Larval body length, (c, d) body width, (e, f) body weight, and (g, h) crawling were measured in control, EtOH, HSD‐fed, HSD + EtOH, and HSD + quercetin (0.25–10 μM) groups. Data are represented as mean ± SD (*p* < 0.0001, *p* < 0.01, ns).

**Figure:**
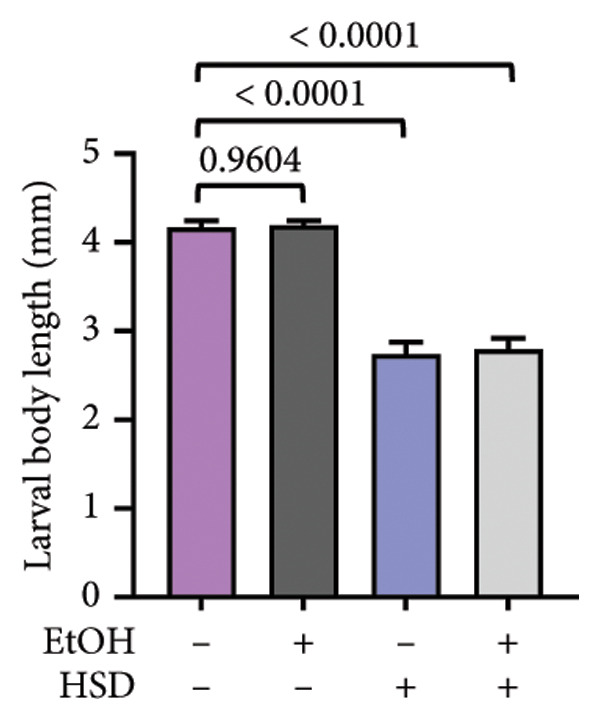
(a)

**Figure:**
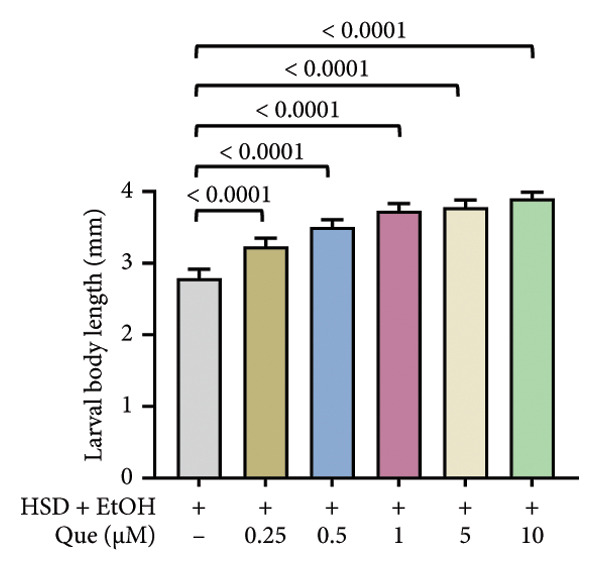
(b)

**Figure:**
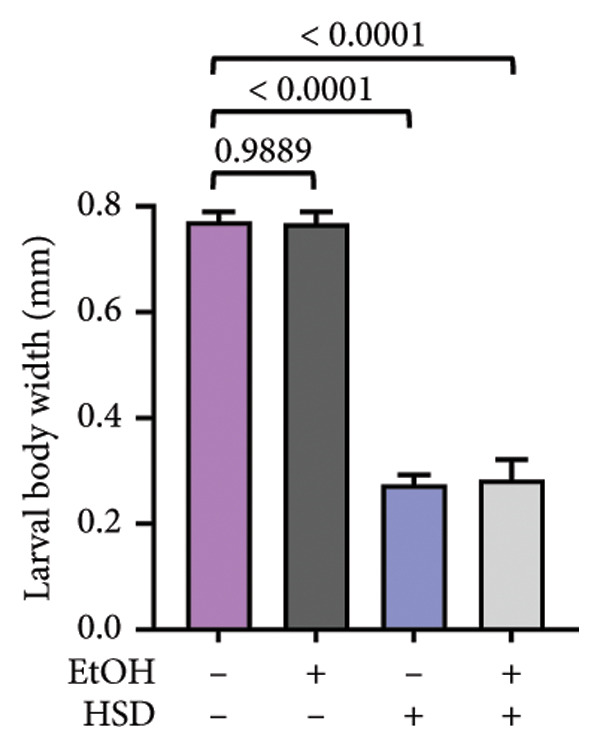
(c)

**Figure:**
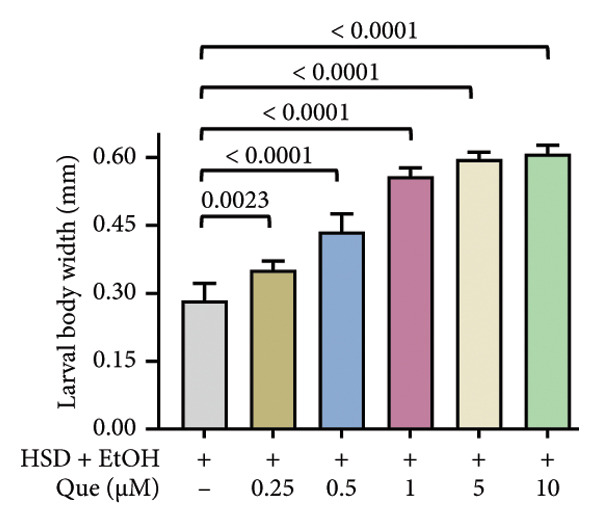
(d)

**Figure:**
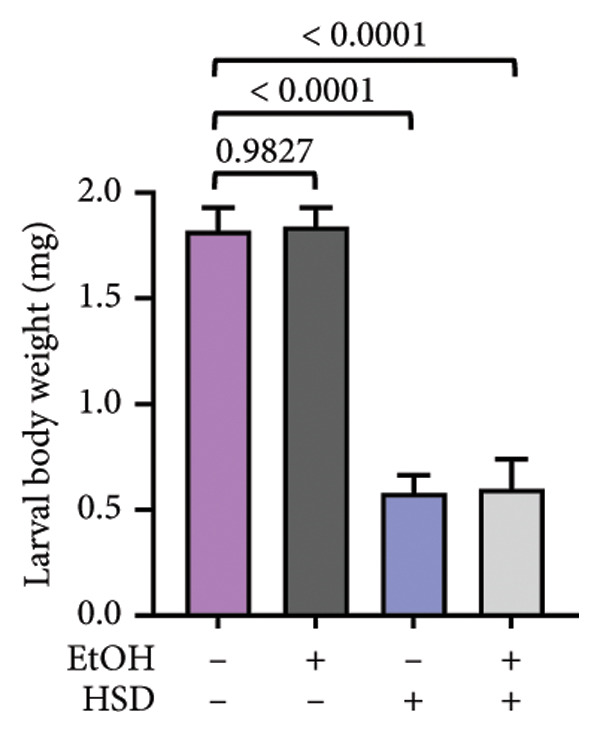
(e)

**Figure:**
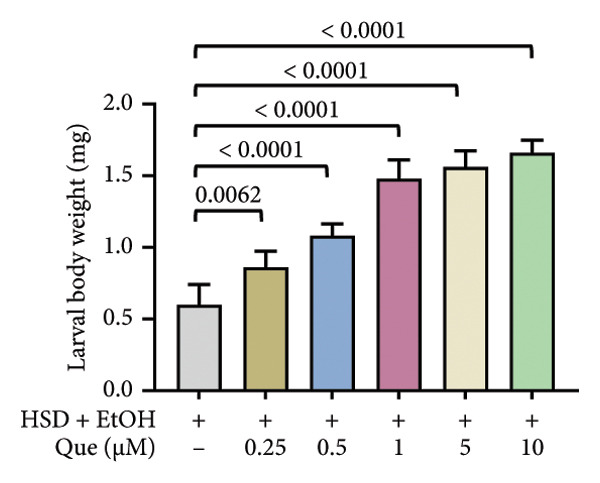
(f)

**Figure:**
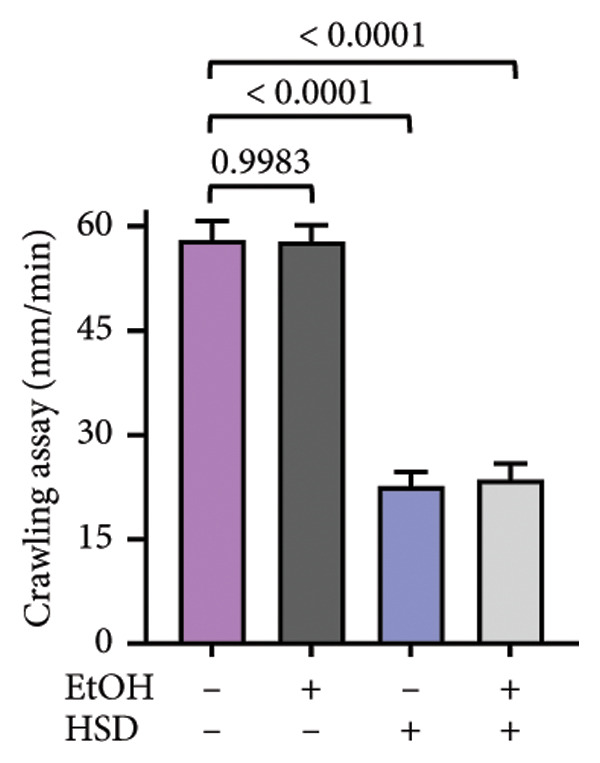
(g)

**Figure:**
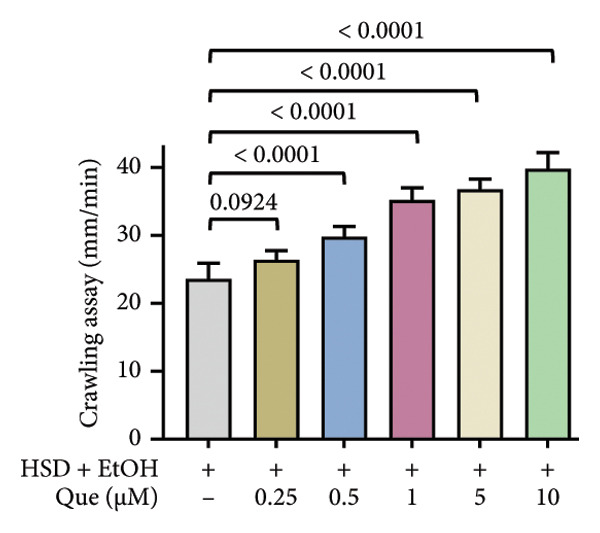
(h)

### 3.2. Quercetin Modulates Hemolymph Glucose and Insulin‐Like Peptide Gene Expression in HSD‐Fed *Drosophila*

The phenotypic impairments observed in *D. melanogaster* larvae, such as reduced body length, width, weight, and motor activity, reflect the significant effects of hyperglycemia induced by an HSD. To further investigate the molecular mechanisms underlying these phenotypic changes, we analyzed hemolymph glucose levels and expression of the insulin‐like peptide gene. Larvae exposed to HSD showed a significant increase in hemolymph glucose compared with the control (Figure 3(a)). Quercetin supplementation markedly reduced glucose levels in HSD‐fed larvae (Figure 3(b)). Expression analysis revealed that *dilp3* transcript levels were significantly downregulated in HSD‐fed larvae relative to the control (Figure 3(c)). Quercetin treatment restored *dilp3* expression toward normal levels (Figure 3(d)). *dilp2* expression also differed significantly among treatments (Figure 3(e)), with quercetin enhancing *dilp2* expression in HSD‐fed larvae. Similarly, *dilp5* expression varied significantly across groups (Figure 3(f)), indicating that quercetin modulates *dilp5* regulation under high‐sugar conditions.

Figure 3Effects of quercetin on hemolymph glucose and insulin‐like peptide expression in *D. melanogaster* exposed to HSD. (a, b) Hemolymph glucose concentration, (c, d) *dilp3*, (e) *dilp2*, and (f) *dilp5* expression relative to *rp49*. Groups include control, EtOH, HSD‐fed, HSD + EtOH, and HSD + quercetin (0.25–10 μM). Data are represented as mean ± SD.

**Figure:**
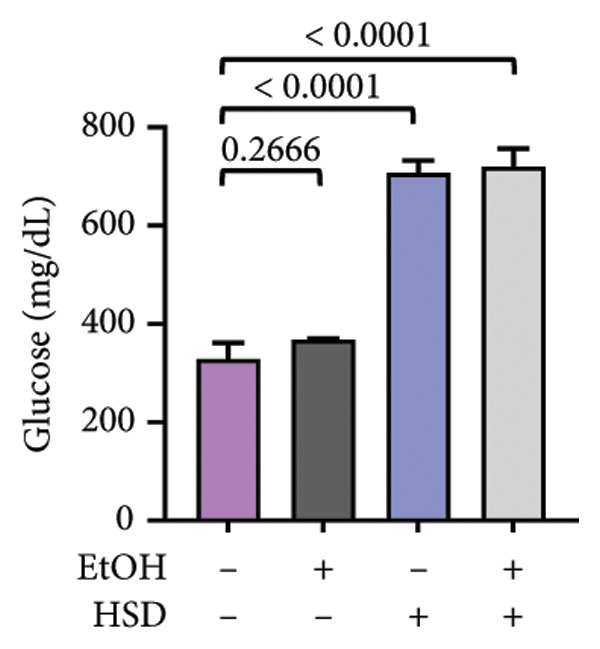
(a)

**Figure:**
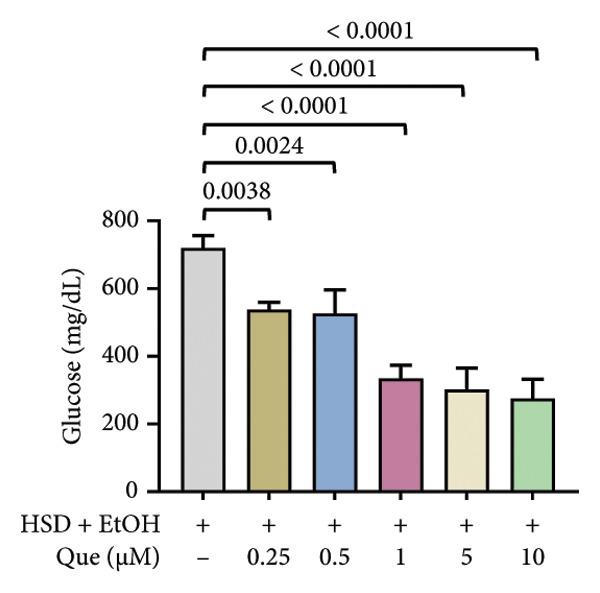
(b)

**Figure:**
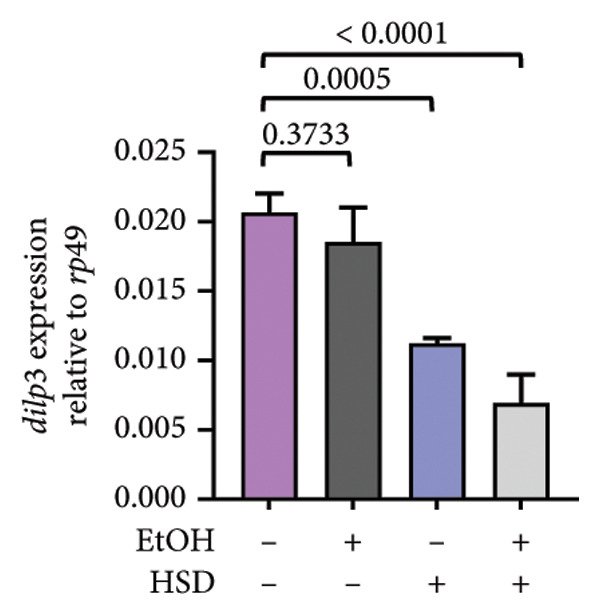
(c)

**Figure:**
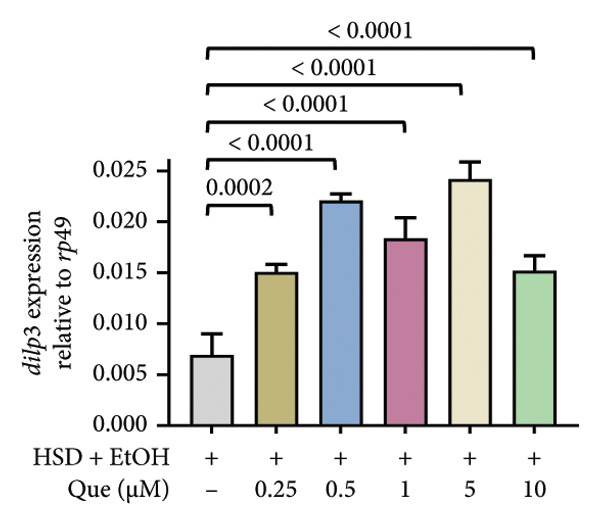
(d)

**Figure:**
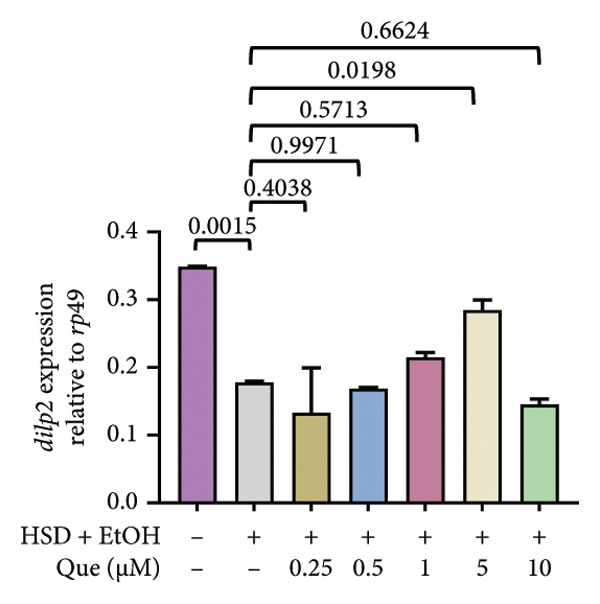
(e)

**Figure:**
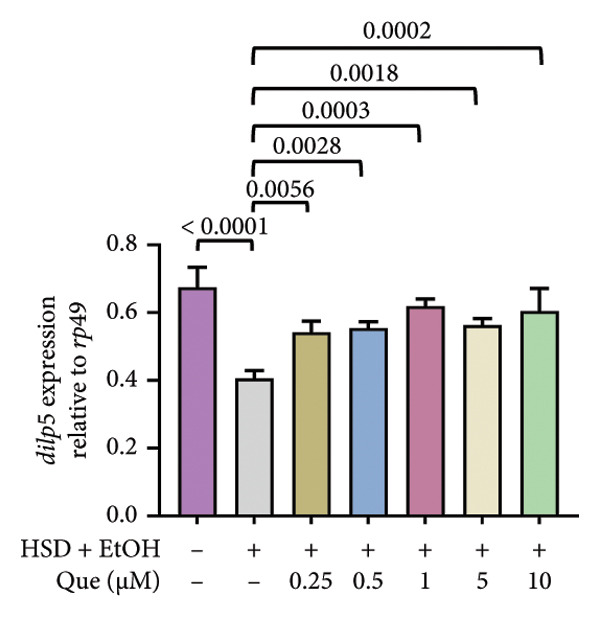
(f)

### 3.3. Quercetin Regulates Metabolic Signaling Response in HSD‐Fed *Drosophila*

To further examine the metabolic signaling response, the expression of *Thor* and *srl* was analyzed. *Thor* expression differed significantly among treatment groups (Figure 4(a)). HSD‐fed larvae showed reduced *Thor* expression compared with the control, while quercetin treatment restored expression levels, particularly at moderate doses. Similarly, *srl* transcript levels varied significantly across treatments (Figure 4(b)). Exposure to HSD decreased *srl* expression relative to control, whereas quercetin partially normalized *srl* levels under HSD conditions.

Figure 4Effects of quercetin on *Thor* and *srl* expression in *D. melanogaster* larvae exposed to HSD. (a) *Thor* and (b) *srl* expression relative to *rp49*. Groups include control, HSD + EtOH, and HSD + quercetin (0.25–10 μM). Data are represented as mean ± SD.

**Figure:**
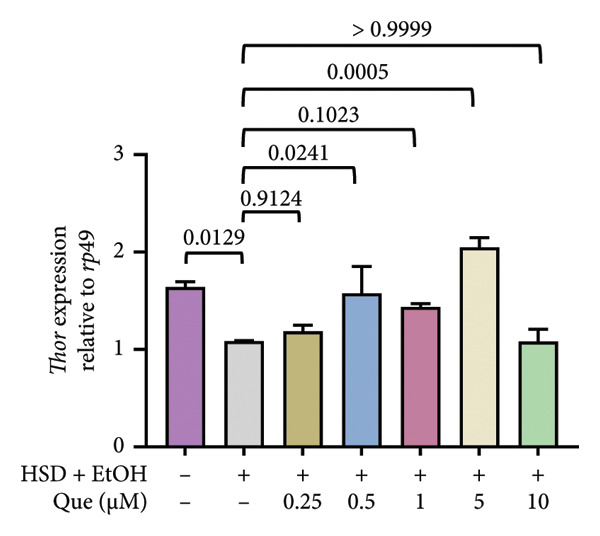
(a)

**Figure:**
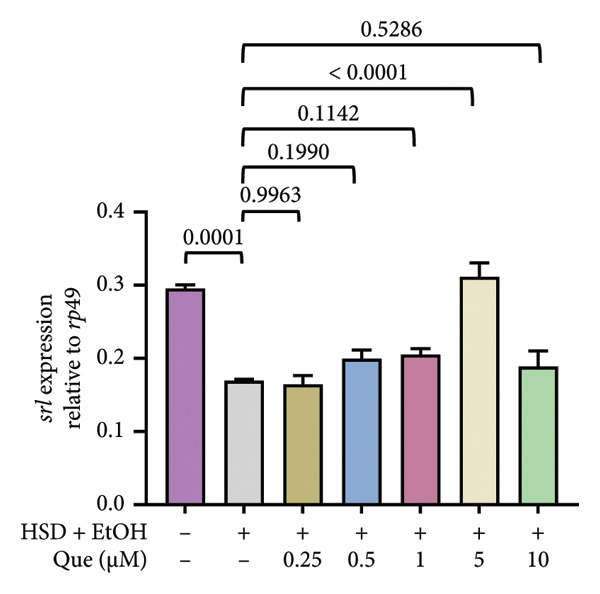
(b)

### 3.4. Quercetin Modulates Stress‐Responsive Gene Expression in HSD‐Fed *Drosophila*

To assess stress‐related responses under HSD conditions, we analyzed the expression of *totA* and *hsp70*. Gene expression level of *totA* differed significantly among treatments (Figure 5(a)), but there was no significant difference between the control and HSD‐fed larvae (Figure 5(b)) Quercetin supplementation markedly increased *totA* expression, particularly at 5 and 10 μM. Meanwhile, *hsp70* expression varied significantly across groups (Figure 5(b)). HSD‐fed larvae showed reduced *hsp70* levels relative to controls, whereas quercetin treatment restored expression toward control levels.

Figure 5Effects of quercetin on stress‐responsive gene expression in *D*. *melanogaster* larvae exposed to HSD. (a) *totA* and (b) *hsp70* expression relative to *rp49*. Groups include control, HSD + EtOH, and HSD + quercetin (0.25–10 μM). Data are represented as mean ± SEM.

**Figure:**
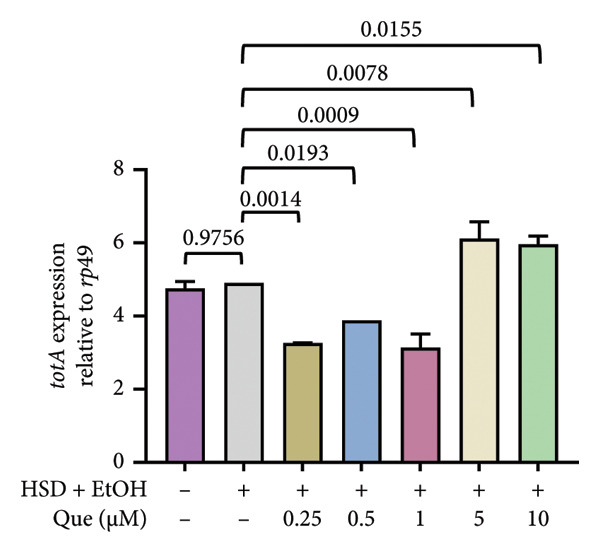
(a)

**Figure:**
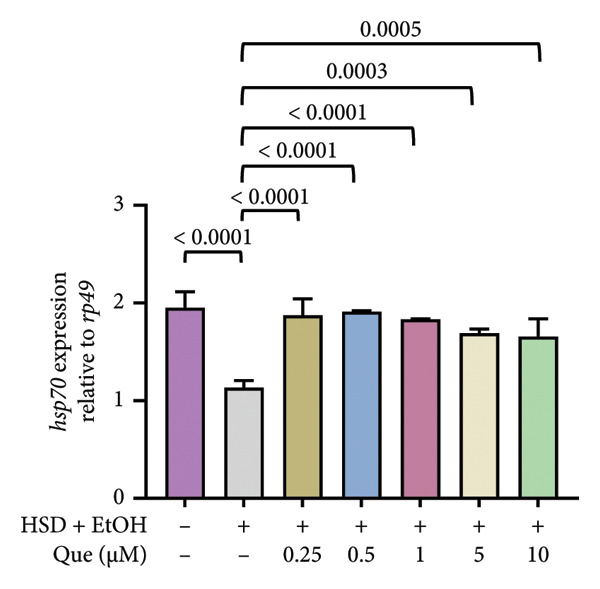
(b)

### 3.5. Quercetin Reduces Hemolymph Glucose Levels Without Affecting Locomotion and Survival in Adult *Drosophila*

A previous study highlighted the potential of adult *D. melanogaster* as a model for screening compounds targeting DPP4 enzymatic activity [29]. Building on these findings, we extended our experiments to adult flies under standard dietary conditions to reevaluate the hypoglycemic potential of quercetin. Adult *D. melanogaster* were treated with quercetin concentrations of 0.5, 1, and 10 μm, and hemolymph glucose levels were measured. Hemolymph glucose levels varied significantly among groups (Figure 6(a)). Quercetin treatment significantly reduced hemolymph glucose levels at 1 and 10 μm concentrations.

In contrast, locomotor performance did not differ significantly among treatments (Figure 6(b)). Similarly, survival rate at day 12 post‐treatment showed no significant difference across groups (Figure 6(c)), indicating that quercetin treatment did not affect adult viability during the observation period.

Figure 6Effects of quercetin on adult *D*. *melanogaster*. (a) Hemolymph glucose concentration, (b) locomotor activity, and (c) survival rate in adult flies. Locomotor ability was assessed using the negative geotaxis assay at days 3, 6, 9, and 12 after treatment, and survival was monitored daily from the start of quercetin administration until day 12. For locomotor and survival assays, each group consisted of 10 flies per replicate, with three replicates per group (*n* = 30 flies per treatment). Data are represented as mean ± SD.

**Figure:**
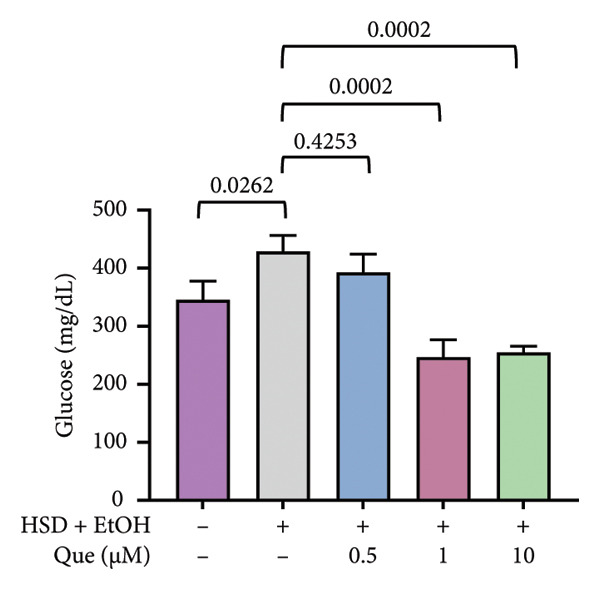
(a)

**Figure:**
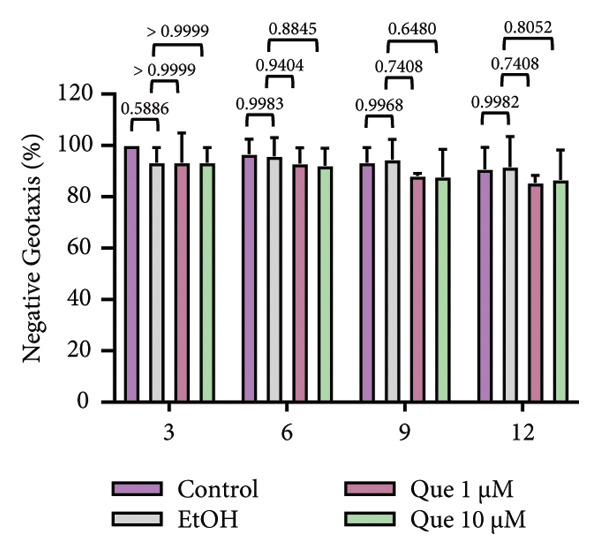
(b)

**Figure:**
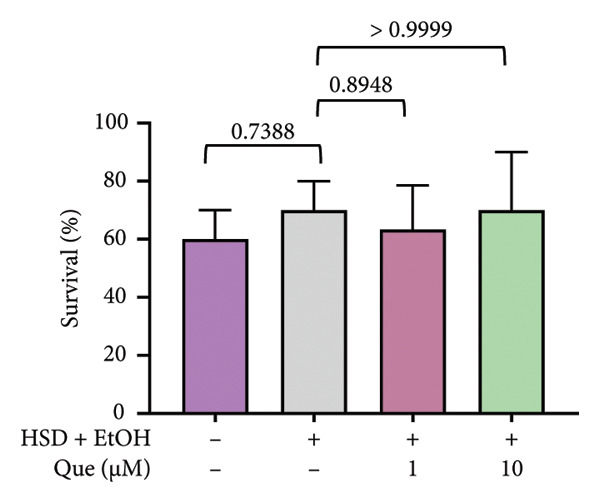
(c)

### 3.6. Quercetin Exhibits High Affinity as a DPP4 Inhibitor Based on Molecular Docking Analysis

**FIGURE 7:**
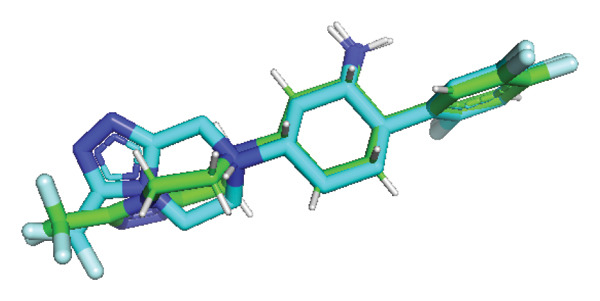
Validation of the docking protocol through redocking of the native ligand into the DPP4 active site, yielding an RMSD value of 0.347 Å.

To further evaluate its potential as a DPP4 inhibitor, a molecular docking analysis was conducted by docking quercetin’s molecular structure into the DPP4 receptor. The objective of this analysis was to compare the binding affinity and interaction profile of quercetin with those of the clinically used DPP4 inhibitor vildagliptin. Prior to docking, the protocol was validated through redocking the native ligand into the DPP4 active site, yielding an RMSD value of 0.347 Å (Figure 7), thereby indicating a reliable and reproducible docking procedure [32].

The docking results revealed that quercetin has a binding energy of −7.8 kcal/mol, which is lower than that of vildagliptin, which exhibits a binding energy of −7.3 kcal/mol. This suggests that quercetin has a higher affinity for the DPP4 receptor. For comparison, the native ligand at the DPP4 active site has the lowest binding energy of −9.8 kcal/mol, which serves as a reference for optimal binding potential. Binding energy values are shown directly in Figures 8(a), 8(b), and 8(c) for clarity.

Further analysis of molecular interactions showed that the native ligand binds to residues Tyr547 through hydrogen bonds (Figure 8(a)). Quercetin, in contrast, forms hydrogen bonds with key active site residues, including Ser630, His740, and Tyr547 (Figure 8(b)). Vildagliptin binds to residues Arg358 and Tyr662 through hydrogen bonds (Figure 8(c).

Figure 8Molecular docking analysis showing ligand interactions with the DPP4 protein. Binding energy values (ΔG) are shown in each panel. (a) Native ligand (ΔG = −9.8 kcal/mol), (b) quercetin (ΔG = −7.8 kcal/mol), (c) vildagliptin (ΔG = −7.3 kcal/mol).

**Figure:**
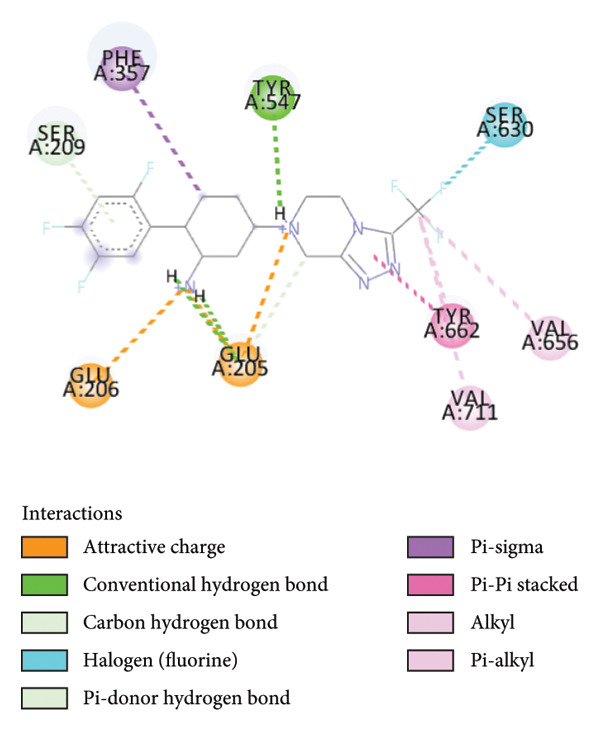
(a)

**Figure:**
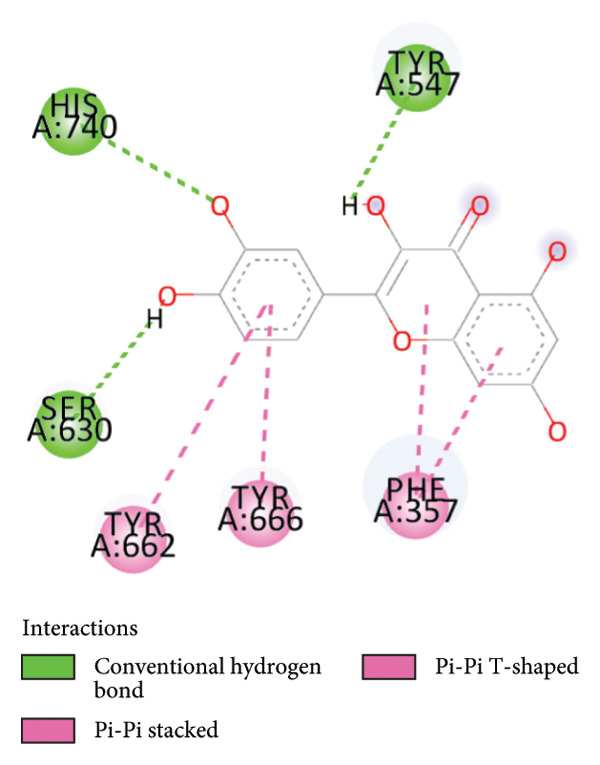
(b)

**Figure:**
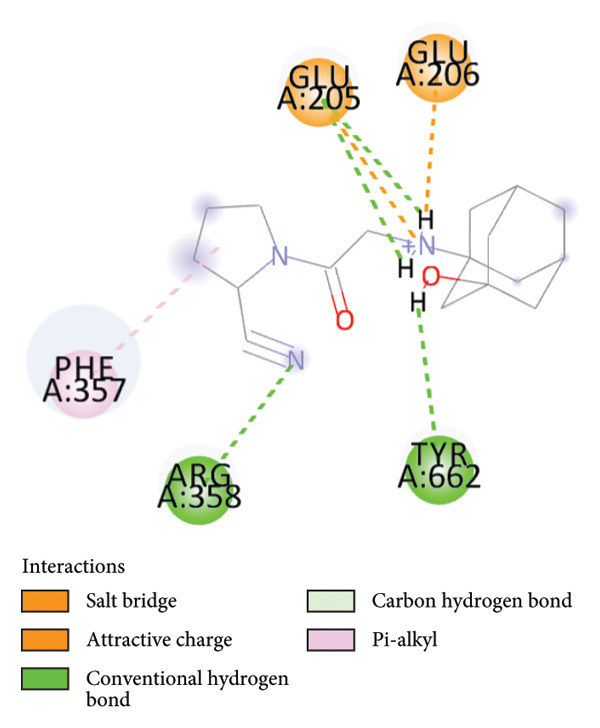
(c)

## 4. Discussion

A previous study has demonstrated that dietary intervention leads to metabolic dysfunction and elevated glucose levels in *D. melanogaster* [24]. In line with this prior evidence, the present study utilized a 30% HSD to establish a hyperglycemic model and evaluate the potential antihyperglycemic properties of quercetin. Quercetin exhibits hypoglycemic activity through mechanisms such as enhancing insulin sensitivity, improving glucose metabolism, and promoting insulin secretion [6, 7]. Based on these properties, the *D. melanogaster* *w* ^1118^strain was utilized across both larval and adult stages to provide a comprehensive assessment of quercetin’s efficacy in mitigating the detrimental effects associated with hyperglycemia.

Under hyperglycemic conditions, phenotypic traits of *D. melanogaster* larvae such as body length, width, weight, and motor activity are often impaired due to metabolic disturbances and oxidative stress [35]. In this study, larvae HSD‐fed exhibited significant reductions in body length (Figures 2(a)) and width (Figures 2(c)), body weight (Figure 2(e)), and crawling activity (Figure 2(g)), consistent with previous findings in hyperglycemic *D. melanogaster* models [28, 35, 36]. These impairments are likely attributed to decreased food intake and reduced energy availability in HSD‐fed larvae [35]. To address these effects, quercetin, a flavonoid known for its strong antioxidant activity, was administered and found to restore several physiological functions by modulating disrupted metabolic pathways [37]. Quercetin has demonstrated therapeutic potential in metabolic disorders, including the regulation of body weight [38], through its role in enhancing cellular energy efficiency and reducing oxidative damage caused by HSD [39]. Accordingly, quercetin treatment led to marked improvements in body length (Figure 2(b)), width (Figure 2(d)), weight (Figure 2(f)), and crawling activity (Figure 2(h)), with the most prominent effect observed at a concentration of 10 μM.

To further validate the antihyperglycemic effect of quercetin, we measured hemolymph glucose levels in the third instar larvae. Hemolymph glucose level in HSD‐fed larvae is significantly increased compared to the control (Figure 3(a)). Meanwhile, quercetin supplementation effectively reduced glucose concentrations in a concentration‐dependent manner, with significant reductions observed starting at 0.25 μM and 0.5 μM, indicating the onset of a threshold response (Figure 3(b)). Higher doses (1–10 μM) exhibit progressively stronger effects (*p* < 0.0001), suggesting that quercetin’s glucose‐lowering activity becomes fully activated beyond this initial threshold range. This pattern implies that quercetin begins to exert metabolic modulation at low micromolar concentrations, likely through the activation of an insulin‐like signaling pathway, which enhances glucose utilization and restores metabolic homeostasis under stress conditions. Therefore, in the subsequent experiment, we investigate the molecular mechanisms through the expression of the insulin‐like peptide genes in *D. melanogaster* larvae.

Gene expression level of *dilp3* (Figures 3(c), 3(d)), *dilp2* (Figure 3(e)), and *dilp5* Figure 3(f)) is markedly decreased in HSD‐fed larvae, reflecting impaired insulin signaling [28, 40]. Quercetin supplementation restored the expression of these insulin‐like peptide genes toward control levels, indicating recovery of insulin pathway function. These results show that quercetin directly alleviates glucose imbalance and normalizes insulin‐related gene expression in *D. melanogaster* larvae under high‐sugar conditions.

In addition to assessing the effect of quercetin in metabolic signaling, we examined the expression of *Thor* (Figure 4(a)) and *srl* (Figure 4(b)). HSD significantly reduced *Thor* expression, indicating suppression of insulin/FOXO downstream signaling [17, 18]. Quercetin treatment restored *Thor* levels, particularly at moderate concentrations. Likewise, *srl* expression, a marker of mitochondrial biogenesis [20], decreased under HSD but was partially normalized by quercetin. These results show that quercetin helps maintain insulin/FOXO activity and mitochondrial function in larvae exposed to an HSD.

Expression of *totA* (Figure 5(a)), a downstream target of the JAK/STAT pathway [21, 22], remained unchanged between the control and HSD‐fed larvae, suggesting that high‐sugar exposure did not strongly activate this stress‐related signaling. However, quercetin supplementation significantly increased *totA* expression, especially at higher doses. In contrast, *hsp70* expression decreased under HSD conditions (Figure 5(b)), reflecting a reduced cellular stress response capacity [23]. Quercetin treatment restored *hsp70* levels toward control values, suggesting improved proteostasis and stress tolerance. Together, these results imply that quercetin mitigates cellular stress imbalances associated with high‐sugar exposure by normalizing the expression of key stress‐regulatory genes.

While previous studies established quercetin’s glucose‐lowering and insulin‐sensitizing effects in both mammals and *D. melanogaster* [6, 38], the present work adds mechanistic depth by examining how quercetin simultaneously modulates insulin‐related (*dilp2, dilp3,* and *dilp5*), metabolic (*Thor* and *srl*), and stress‐associated (*totA* and *hsp70*) genes under high‐sugar conditions. This integrated response suggests that quercetin restores metabolic homeostasis through coordinated regulation of metabolic signaling and stress resilience rather than acting solely as a glucose‐lowering compound.

Building on previous findings that identified adult *D. melanogaster* as a suitable model for screening compounds targeting DPP4 enzymatic activity [29], this study further evaluated the hypoglycemic potential of quercetin in adult flies under standard dietary conditions. Notably, significant decreases in glucose levels were recorded at 1 μM and 10 μM (Figure 6(a)), indicating quercetin’s potential role as a hypoglycemic agent, possibly via DPP4 inhibition (Figure 6(a)) [38, 41]. To ensure that glucose‐lowering effects did not compromise motor function, a negative geotaxis assay was performed. Locomotor activity, a key indicator of neuromuscular health in *D. melanogaster* [42, 43], showed no significant differences between treated and control groups across 3, 6, 9, and 12 days, indicating that quercetin did not impair motor function (Figure 6(b)). Additionally, a 12‐day survival analysis was conducted to assess potential toxicity. No significant differences in survival rates were observed between the groups, even at the highest concentration (10 μM), suggesting that quercetin is nontoxic and safe at the tested doses (Figure 6(c)). The 12‐day observation period was chosen as it represents approximately 15% of the fly’s average lifespan (80–100 days) [44], making it sufficient to evaluate the acute to subchronic effects of quercetin, including glucose regulation and potential side effects. Overall, these findings demonstrate that quercetin effectively lowers blood glucose levels in adult *D. melanogaster* without negatively impacting locomotor ability.

Molecular docking is a widely used computational approach for predicting and characterizing ligand–protein binding interactions [33]. In docking analyses, a more negative binding free energy indicates a stronger predicted ligand–protein affinity [34]. For DPP4, key residues within the catalytic and substrate‐binding site include Ser630, Arg125, Tyr547, and Arg350 [34, 45]. Quercetin exhibited a binding energy of −7.8 kcal/mol, lower than that of vildagliptin −7.3 kcal/mol, suggesting a higher predicted affinity for DPP4. The native ligand of DPP4 used as a reference showed the strongest binding energy at −9.8 kcal/mol. Interaction analysis revealed that the native ligand formed hydrogen bonds with Tyr547 (Figure 8(a)). Quercetin binds to Ser630, His740, and Tyr547 (Figure 8(b)), while vildagliptin interacts with Arg358 and Tyr662 (Figure 8(c)). Notably, quercetin engaged Ser630 and Tyr547, residues that are critical for DPP4 catalytic activity, and shared interactions with the native ligand. These results indicate that quercetin binds effectively within the DPP4 active site and may function as a potent DPP4 inhibitor, exhibiting a predicted binding affinity comparable to or potentially exceeding that of vildagliptin.

Collectively, these in vivo and in silico results highlight the potential of *D. melanogaster* as a robust model for screening antihyperglycemic candidates targeting metabolic pathways. A recent study has likewise highlighted the utility of Drosophila as a screening platform for identifying bioactive compounds with DPP4 inhibitory activity [29]. By integrating phenotypic, molecular, and computational endpoints, the present study aligns with these emerging methodologies and further reinforces the relevance of *D. melanogaster* for large‐scale drug discovery.

Although quercetin is widely recognized for its beneficial effects on glucose homeostasis and the attenuation of oxidative stress, it has also been reported to exert cytotoxic and anticancer effects at higher concentrations [8]. In this study, quercetin exhibited antihyperglycemic activity without detectable impairments in survival or behavior, suggesting that the tested concentrations (0.25–10 μM) were nontoxic under the experimental conditions. Nevertheless, dedicated cytotoxicity and genotoxicity assessments were not performed and should be incorporated in future studies (e.g., MTT, comet, or micronucleus assays) to more precisely define the safety profile and enhance the translational relevance of these findings.

While *D. melanogaster* offers a powerful and ethically accessible model for studying metabolic regulation and pharmacological effects, translation of these findings to mammalian systems should be undertaken with caution. The conservation of insulin signaling, metabolic control, and stress‐response pathways supports the relevance of this model for preliminary screening. However, species‐specific differences in absorption, metabolism, and the potential cytotoxicity of quercetin necessitate further validation in mammalian or human‐derived systems to confirm efficacy, define optimal dosing, and establish safety for diabetes management.

## 5. Conclusion

This study demonstrates that quercetin exerts significant hypoglycemic effects in *D. melanogaster*, as evidenced by reduced hemolymph glucose levels, modulation of metabolic‐ and stress‐response gene expression, and improvements in larval growth and locomotor performance under high‐sugar conditions. These in vivo findings are supported by molecular docking analyses indicating a strong predicted binding affinity for DPP4, along with the absence of detectable adverse effects on adult survival or locomotor activity. Collectively, the results provide preliminary evidence that quercetin represents a promising phytochemical candidate for further development as an antidiabetic agent. Future studies should validate these effects in mammalian models, with particular emphasis on comprehensive cytotoxicity assessment and dose‐escalation safety studies to strengthen translational relevance.

## Conflicts of Interest

The authors declare no conflicts of interest.

## Funding

This study was not supported by external funding.

## Data Availability

All data generated or analyzed during this study are included in this manuscript.
